# Computational screening for potential drug candidates against the SARS-CoV-2 main protease

**DOI:** 10.12688/f1000research.23829.2

**Published:** 2020-12-21

**Authors:** Bruno Silva Andrade, Preetam Ghosh, Debmalya Barh, Sandeep Tiwari, Raner José Santana Silva, Wagner Rodrigues de Assis Soares, Tarcisio Silva Melo, Andria Santos Freitas, Patrícia González-Grande, Lucas Sousa Palmeira, Luiz Carlos Junior Alcantara, Marta Giovanetti, Aristóteles Góes-Neto, Vasco Ariston de Carvalho Azevedo

**Affiliations:** 1 Laboratório de Bioinformática e Química Computacional, Departamento de Ciências Biológicas, Universidade Estadual do Sudoeste da Bahia (UESB), Jequié, Bahia, 45205-490, Brazil; 2 Department of Computer Science, Virginia Commonwealth University, Richmond, VA, 23284, USA; 3 Centre for Genomics and Applied Gene Technology, Institute of Integrative Omics and Applied Biotechnology (IIOAB), Purba Medinipur, India; 4 Laboratório de Genética Celular e Molecular, Departamento de Biologia Geral, Instituto de Ciências Biológicas, Universidade Federal de Minas Gerais, Belo Horizonte, Minas Gerais, MG, Brazil; 5 Programa de Pós-graduação em Genética e Biologia Molecular, Universidade Estadual de Santa Cruz, Ilhéus, BA, Brazil; 6 Departamento de Saúde II, Universidade Estadual do Sudoeste da Bahia., Jequié, BA, Brazil; 7 Laboratório de Flavivírus, Instituto Oswaldo Cruz, Fundação Oswaldo Cruz, Rio de Janeiro, RJ, Brazil; 8 Laboratório de Biologia Molecular e Computacional de Fungos, Departamento de Microbiologia, Instituto de Ciências Biológicas, Universidade Federal de Minas Gerais (UFMG), Belo Horizonte, MG, Brazil

**Keywords:** SARS-CoV-2, protease, virtual screening, pharmacophore, inhibitors, natural compounds

## Abstract

**Background:** SARS-CoV-2 is the causal agent of the current coronavirus disease 2019 (COVID-19) pandemic. They are enveloped, positive-sense, single-stranded RNA viruses of the Coronaviridae family. Proteases of SARS-CoV-2 are necessary for viral replication, structural assembly, and pathogenicity. The approximately 33.8 kDa M ^pro^ protease of SARS-CoV-2 is a non-human homologue and is highly conserved among several coronaviruses, indicating that M ^pro^ could be a potential drug target for Coronaviruses.

**Methods:** Herein, we performed computational ligand screening of four pharmacophores (OEW, remdesivir, hydroxychloroquine and N3) that are presumed to have positive effects against SARS-CoV-2 M ^pro ^protease (6LU7), and also screened 50,000 natural compounds from the ZINC Database dataset against this protease target.

**Results:** We found 40 pharmacophore-like structures of natural compounds from diverse chemical classes that exhibited better affinity of docking as compared to the known ligands. The 11 best selected ligands, namely ZINC1845382, ZINC1875405, ZINC2092396, ZINC2104424, ZINC44018332, ZINC2101723, ZINC2094526, ZINC2094304, ZINC2104482, ZINC3984030, and ZINC1531664, are mainly classified as beta-carboline, alkaloids, and polyflavonoids, and all displayed interactions with dyad CYS145 and HIS41 from the protease pocket in a similar way as other known ligands.

**Conclusions:** Our results suggest that these 11 molecules could be effective against SARS-CoV-2 protease and may be subsequently tested *in vitro* and *in vivo* to develop novel drugs against this virus.

## Introduction

Coronaviruses (CoVs) are enveloped, positive-sense, single-stranded RNA viruses of the Coronaviridae family ^1^. Based on their antigenic properties, they were classified into three main groups ^2^: i) alpha-CoVs, responsible for gastrointestinal disorders; ii) beta-CoVs, which include: (a) Bat coronavirus (BCoV), (b) human severe acute respiratory syndrome (SARS) virus, (c) Middle Eastern respiratory syndrome (MERS) virus; and iii) gamma-CoVs, which mainly infect avian species. The most well-known of these coronaviruses is the SARS-CoV, responsible for causing an outbreak in 2002–2003 ^3^ and MERS-CoV, causing severe respiratory symptoms, which was identified in 2012 ^4^.

In December 2019, a series of unusual pneumonia cases caused by a novel coronavirus, recently renamed as SARS-CoV-2, was identified in Wuhan, China ^5– 7^. The disease caused by SARS-CoV-2 is now called COVID-19, and displays vast pathophysiological aspects, which include symptoms, such as fever and coughing, and severe acute respiratory failure ^8^. Since the infection crossed geographical barriers, the World Health Organization (WHO) declared a pandemic situation in March 2020, reaching a worldwide mortality rate of approximately 3% ^6^.

The SARS-CoV-2 ORF1ab code for polyprotein 1ab (pp1ab), where the main protease M ^pro^ is found, which is similar to a key enzyme in the processing of the picornavirus family polyprotein. The protease M ^pro^, digests more than 11 conserved sites starting from its autolytic cleavage in pp1ab, and is a protein with extreme functional importance in the viral cycle ^9^. Due to its great importance in the coronavirus cycle, the M ^pro^ sequence of SARS-CoV-2 shows more than 90% similarity with the enzymes of other coronaviruses ^10^ and shares 96% identity with SARS-CoV. Although M ^pro^ is conserved among SARS-CoVs, it has a loop that can make it difficult for an inhibitor to access the catalytic pocket, and mutations in this loop can generate drug resistance ^11^. Thus, even though M ^pro^ is one of the most conserved SARS-CoV group proteins, point mutational aspects can lead to a possible drug resistance, so that a wide range of inhibitor options is necessary for the treatment of COVID-19.

ORF1ab is characteristic of members of the Coronaviridae family ^12^ and is equivalent to two-thirds of the SARS-CoV-2 virus genome ^13^. Each of these ORFs encodes a polyprotein (pp), which, when cleaved by proteases contained in the sequence, will generate 11 proteins (pp1a) and 5 proteins (pp1ab), respectively. The functions associated with these proteins are related to the virus replication processes and the modulation of the immune response in the host, among other essential functions for the development of the pathogen within the host cell ^6^.

Virus resistance to drugs can lead to the emergence of new epidemics, such as influenza A virus (IAV). In this case, two drug classes have been related: M2 channel inhibitors (amantadine and rimantadine) and neuraminidase inhibitors (NAIs; oseltamivir, zanamivir, peramivir, and Laninamivir). Both drug classes act by inhibiting proteins that are located in the viral envelope, and this region is in greater contact with the external environment and is prone to suffer from greater evolutionary pressure and, consequently, mutations ^14^. Drug resistance can occur when rapid viral replication is not repressed completely ^15^. In contrast, virus proteases play a crucial role during virus replication and, therefore, they are a good target for drug discovery ^16^.

During viral replication, proteases are necessary for the assembly of the viral structure, and there have already been suggested to have relationships with the mechanism of infection and pathogenicity of SARS-CoV-2 ^5, 17^. Proteases are enzymes found in all cellular organisms and viruses and are classified according to their catalytic nature. Proteases are divided into four groups: serine, cysteine, aspartyl and metalloproteases. Different types of proteases can perform the same activity through different catalytic mechanisms ^16^, and a protease commonly has a binding site and a catalytic site that are very close in the protein structure ^16^. Furthermore, proteases are present in several types of viruses and are widely found in human viruses ^18^.

In coronaviruses, pp1 is essential for the replication of the virus, as it encodes the protease M ^pro^, which is also called the “main protease” ^19, 20^. M ^pro^ is classified as a chymotrypsin-like cysteine protease (3CLpro), EC: 3.4.19.12, ^10, 19^, and the M ^pro^ protease of SARS-CoV-2, which has a mass of approximately 33.8 kDa ^20^, is characterized by a self-cleavage protein ^21, 22^. It consists of a homodimer subdivided into two protomers (A and B) that have three distinct domains ^23^. The first and second domains have antiparallel β-sheets while the third domain contains five α-helices forming a globular group, which is connected in parallel with the domain-II through a loop region ^20^. The M ^pro^ of SARS-CoV-2 has a catalytic cleft, consisting of a Cys-His dyad in the place of the protease substrate interaction, which is situated between domains -I and -II ^20^. It also has non-canonic specificity to the substrate in the C-terminal portion. Furthermore, there is no homologue of M ^pro^ in the human genome ^20, 24^, and it is highly conserved amongst coronaviruses ^25^. Therefore, M ^pro^ is a potential target for studying inhibitors.

Antiviral therapy considers three main approaches for the control and avoidance of viral infections: (a) vaccination, (b) stimulation of host resistance mechanisms, and (c) antiviral chemotherapy. Antivirals are drugs that inhibit certain virus-specific events, such as binding to host cells, which is how SARS-Cov-2 binds to ACE2 and TMPRSS2 ^26^, and MERS binds to the DPP415 receptor ^27^. Antiviral chemotherapy can involve interfering with any or all of these viral replication steps. Most antiviral drugs are primarily targeted to the synthesis of nucleic acids in viruses. As viral replication and host cell processes are closely linked, one of the main problems of viral therapy would be to find a drug capable of being selectively toxic only for the virus. Antivirals are frequently more effective in prevention than in the treatment itself, and are ineffective in eliminating latent or non-replicating viruses ^28^. In addition, when selecting an antiviral drug, viral resistance must also be considered since it is one of the main causes of therapeutic failure.

The main classes of antiviral drugs used in clinical therapy to treat systemic viral infections include: a) synthetic nucleosides (e.g. acyclovir, famciclovir, ganciclovir, valacyclovir, and valganciclovir; b) pyrophosphate analogs (e.g. foscarnet); c) drugs for syncytial virus and influenza A (e.g. amantadine and rimantadine hydrochloride and ribavirin); d) nucleoside reverse transcriptase inhibitors (NRTI; e.g. abacavir, didanosine, emtricitabine, stavudine, lamivudine, zidovudine, tenofovir in combination with NRTI); e) non-nucleoside reverse transcriptase inhibitors (NNRTI; e.g. delavirdine, efavirenz, nevirapine); and f) protease inhibitors (e.g. amprenavir, atazanavir, darunavir, fosamprenavir, lopinavir and ritonavir, nelfinavir mesylate, saquinavir mesylate, ritonavir, indinavir sulfate and tipranavir) ^7, 29– 31^

Computational studies of inhibitors that may reduce viral replication is a fast way for proposing drug candidates that can contribute to a reduction in severity and spread of the disease. Moreover, the use of antiviral compounds can assist in the prophylaxis of SARS-CoV-2 and reduce its spread ^32^. Therefore, screening for potential viral protease inhibitors may assist in the selection of new drugs with antiviral potential for SARS-CoV-2.

## Methods

### Ligand screening

For this study, we employed both ligand-based virtual screening (LBVS) and receptor-based virtual screening (RBVS) approaches, considering 50,000 structures of natural compounds from the ZINC Database, which has more than 900 million structures deposited, and includes millions of drug-like compounds that can be obtained for *in vitro* and *in vivo* tests ^33^. The ZINC molecules that were downloaded were those restricted to absorption, distribution, metabolism, excretion and toxicity characteristics (ADMET) for drug likeness: no more than 5 hydrogen bond donors, no more than 10 hydrogen bond acceptors, molecular weight between 160 and 500 Daltons and logP between -0.4 and 5.6 ^34, 35^. For LBVS, we defined four known drugs divided in the following groups: 1) peptide-like crystallographic ligands (N3 and OEW); and 2) repurposed drugs (remdesivir (nucleoside) and hydroxychloroquine) for chemical comparison with our database.

Crystallographic ligand structures were obtained from their corresponding PDB files 6LU7 (N3) and 6Y7M (OEW). Additionally, these structures were used for re-docking validations. In the LBVS process, we used a simple run with vROCS (OpenEye) ^36^ for generating queries with the pharmacophoric map with the stereochemical characteristics for each known ligand. Another option for pharmacophore generation and searching is the free software PharmaGist ^37^. Afterwards, we submitted each ligand query for searching similar pharmacophore-like molecules using the Tanimoto Combo algorithm ^38, 39^ with a cutoff of 1.0, which returned the best 1,000 hits for each round. This procedure was repeated three times for each query, and, subsequently, redundant structures were discarded, generating, in the end, a total of 4,000 similar molecules for the docking experiment.

### Docking studies

Considering PDB validation indices as crystallographic resolution, Ramachandran outliers, clash score, and release date, we selected the structure 6LU7 for RBVS, which is complexed with the peptide-like inhibitor N3 ^20^. Furthermore, 6LU7 and 6Y7M ^40^ were used for re-docking validations with its corresponding crystallographic inhibitors.

The best LBVS hits were submitted to molecular docking calculations with 6LU7 structure using Autodock 4.2 virtual screening protocol ^41^. Ligand structures were prepared for virtual screening using Raccoon plugin ^42^ for Autodock Tools ^42^ according to the standard protocol ^39^, as well as the 6LU7 structure. The gridbox was defined on the active site region, considering the amino acids THR 190, GLU 166, GLN 189, GLY 143, HIS 163, HIS 164, CYS 145, PHE 140, and with accordance with previous studies with the crystallographic structure of the SARS-CoV-2 main protease ^20, 40, 43^. Each docking run was performed three times using the following specifications: flexible docking and Lamarckian Genetic Algorithm with 2,500,000 generations. Afterwards, the 10 best docking hits were selected using the Autodock Tools script *summarize_results4.py*, which can classify the best hits according to their lowest energy clustering conformations and root mean square deviation (RMSD) values. The results were organized according to the ligand pharmacophore relationship with the known structures in Table 2. Docking and re-docking results were evaluated at each docking position inside the 6LU7 active site using Pymol 2.1 ^44^ and UCSF Chimera 1.14 ^45^ in order to confirm molecule interactions with the amino acids within the protease active site. Furthermore, 2D interaction maps were generated by Discovery Studio 2019 ^46^. Another option for 2D map generation is the LigPlot+ software ^47^.

## Results

### LBVS

**Figure 1.**
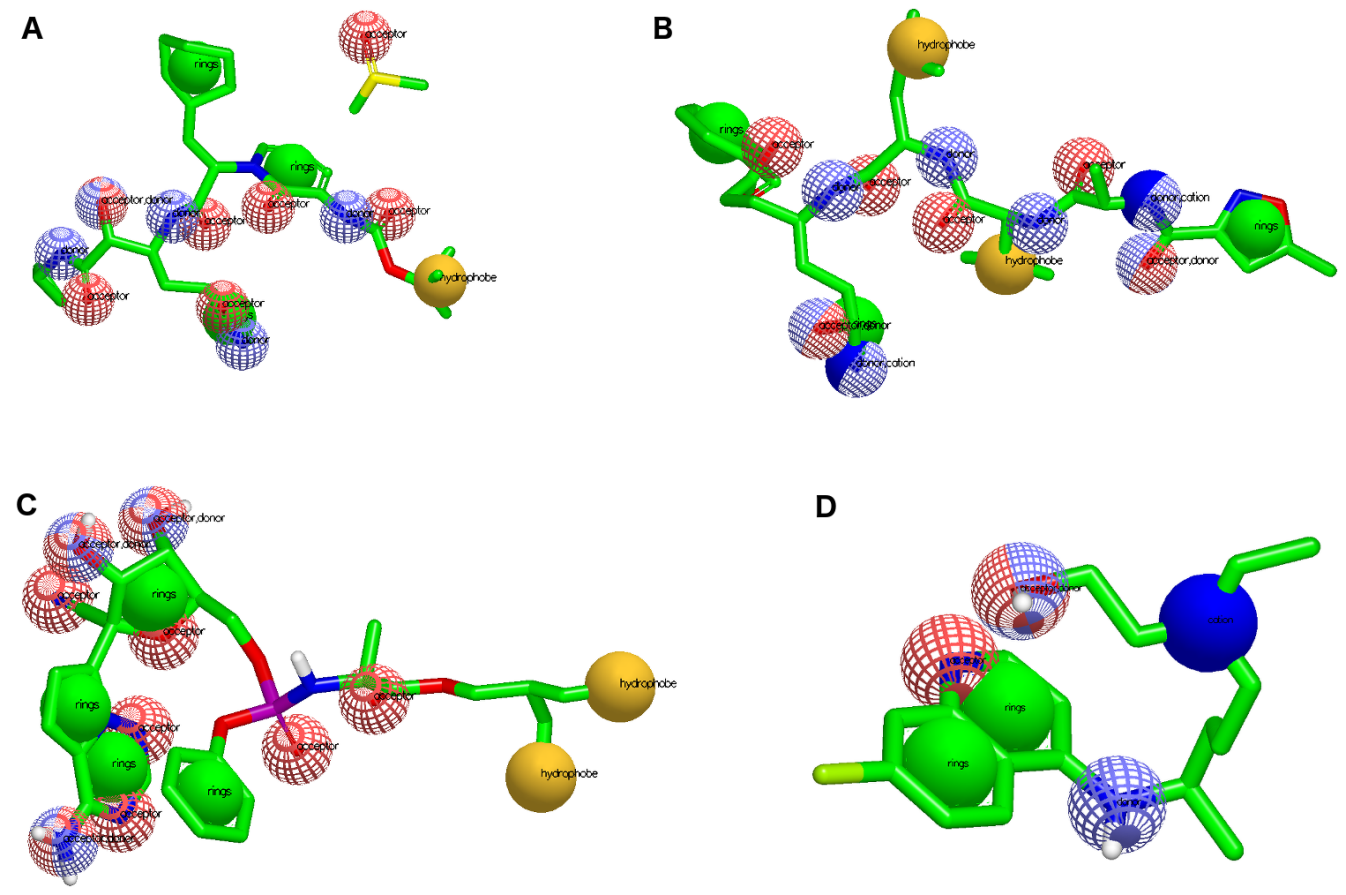
Pharmacophore representation for each known drug used for virtual screening. ( **A**) OEW, ( **B**) N3, ( **C**) remdesivir and ( **D**) hydroxychloroquine. In red spheres: hydrogen acceptors; blue spheres: hydrogen donors; yellow spheres: hydrophobic; and green spheres: aromatic.

Different pharmacophoric characteristics were generated for each known ligand ( Figure 1), which allowed us to find molecules included in different chemical classifications and amplify the number of possible drug candidates. Table 1 shows the pharmacophoric characteristics for each known 6LU7 inhibitor, which permitted us to find natural ligands with pharmacophore-like regions. Additionally, we used the ADMET characteristics for molecular weight and LogP that are important for molecular druggability.

**Table 1.** Pharmacophoric characteristics for each known inhibitor used for screening natural ligands from the ZINC Database.

Inhibitor	Hb.A.	Hb.D.	Aromatic	Hydrophobic	M.W.	LogP
OEW	7	5	3	1	663.8	-0.71
N3	6	4	3	2	680.8	2.32
Remdesivir	9	1	4	2	602.6	1.9
Hydroxychloroquine	2	2	2	0	335.9	3.6

Hb.A. = hydrogen acceptor; Hb.D. = hydrogen donor; M.W. = molecular weight.

Ligand based virtual screening and docking calculations of ZINC database compounds revealed the 40 best pharmacophore-like ligands that belong to different chemical classes, namely beta-carboline alkaloids, indole alkaloids, lupin alkaloids, harmala alkaloids, polyflavonoids, anthracenes, angular pyranocoumarins, and flavonoid-3-O-glycosides. Table 2 shows the detailed results on the average affinity energies, ZINC identification, and chemical classification of each selected ligand.

**Table 2.** The 40 best molecule hits of COVID-19 main protease inhibitor candidates from a dataset of 50,000 natural compounds from the ZINC Database.

Known Drug	Ligand	Energ. Binding (Kcal/Mol)	Classification	RMSD Å	Pred. IC50 (uM)	Exp. IC50 (uM)	Tanimoto combo
OEW	OEW *	-8.86	Peptide-like	1.97	0.320	0.670	-
	ZINC1845382	-10.2	β-carboline Alkaloid				1.12
	ZINC1875405 **	-10.1	β-carboline Alkaloid				1.00
	ZINC2092396	-9.8	β-carboline Alkaloid				1.20
	ZINC1900463	-9.8	β-carboline Alkaloid				1.00
	ZINC2149492	-9.8	β-carboline Alkaloid				1.12
	ZINC2112405	-9.7	β-carboline Alkaloid				1.10
	ZINC2095426	-9.7	β-carboline Alkaloid				1.00
	ZINC2094306	-9.6	β-carboline Alkaloid				1.00
	ZINC2144677	-9.6	Anthracene				1.10
	ZINC1095868	-9.5	Harmala Alkaloids				1.13
	N3 *	-9,77	Peptide-like	1.94	0.07	4.67	-
N3	ZINC2104482	-10.1	β-carboline Alkaloid				1.10
	ZINC3984030	-9.9	Polyflavonoid				1.20
	ZINC1531664	-9.8	Polyflavonoid				1.10
	ZINC2152199	-9.8	β-carboline Alkaloid				1.00
	ZINC4096847	-9.6	Flavonoid-3-O- glycoside				1.20
	ZINC3947428	-9.6	Flavonoid-3-O- glycoside				1.20
	ZINC2092587	-9.6	β-carboline Alkaloid				1.14
	ZINC2115924	-9.5	β-carboline Alkaloid				1.00
	ZINC2110081	-9.5	Lupin Alkaloid				1.00
	ZINC1898165	-9.5	Benzofuran				1.00
	HCQ	-7.90	4-aminoquinoline				-
Hydroxychloroquine (HCQ)	ZINC2101723	-10.2	β-carboline Alkaloid				1.10
	ZINC2094526	-9.8	β-carboline Alkaloid				1.14
	ZINC2094304	-9.6	β-carboline Alkaloid				1.16
	ZINC2091604	-9.4	β-carboline Alkaloid				1.00
	ZINC2113496	-9.4	β-carboline Alkaloid				1.10
	ZINC1460216	-9.3	Angular Pyranocoumarin				1.12
	ZINC2123008	-9.2	β-carboline Alkaloid				1.10
	ZINC682759	-9.2	Harmala Alkaloids				1.00
	ZINC2105243	-9.2	β-carboline Alkaloid				1.00
	ZINC2111696	-9.1	β-carboline Alkaloid				1.20
	REMD	-8.28	Nucleoside				-
Remdesivir (REMD)	ZINC2104424	-10.6	β-carboline Alkaloid				1.15
	ZINC1875405 **	-10.1	β-carboline Alkaloid				1.22
	ZINC44018332	-10.0	Polyflavonoid				1.20
	ZINC2148932	-9.9	β-carboline Alkaloid				1.00
	ZINC2156531	-9.9	Indoles Alkaloid				1.00
	ZINC3197535	-9.9	Polyflavonoid				1.00
	ZINC2102620	-9.9	Indoles Alkaloid				1.00
	ZINC2123402	-9.9	β-carboline Alkaloid				1.00
	ZINC2149488	-9.9	β-carboline Alkaloid				1.10
	ZINC1531664	-9.9	Polyflavonoid				1.00

*Re-docked crystallographic structures.

**Repeated ligand between OEW and Remdesivir pharmacophores.

**Figure 2.**
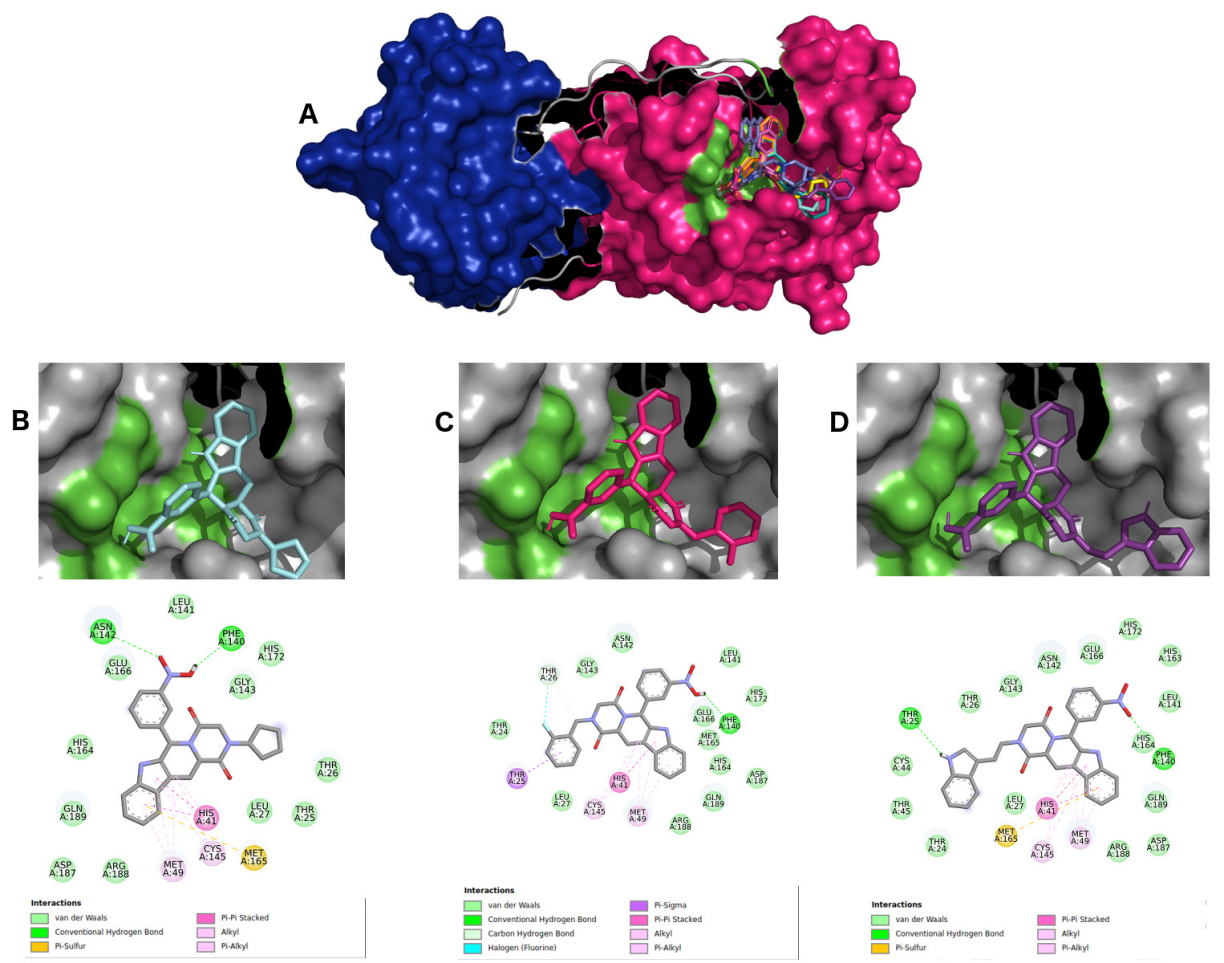
( **A**) SARS-CoV-2 main protease complexed with the 10 best hits for OEW pharmacophore molecules. Protomer A is represented in marine blue surface and protomer B in dark pink surface. ZINC1845382 in cyan ( **B**), ZINC1875405 in dark pink ( **C**) and ZINC2092396 in purple ( **D**) inside 6LU7 binding site and their 2D interaction maps with pocket amino acids.

For selecting the best pharmacophore-like drug-candidates, we considered evaluating lower affinity energy values, as well as interactions with residues of the active site within the target. As can be seen in Figure 2A, all pharmacophore-like OEW ligand molecules formed a complex with the active pocket of 6LU7. The three best OEW ligands (ZINC1845382, ZINC1875405, ZINC2092396) are shown in complex with COVID-19 protease in Figure 2B–D with the detailed 2D interaction map. In this case, these top three hits are included in the beta-carboline alkaloid class.

### RBVS

The intermolecular interactions carried out by ligand ZINC1845382 exhibited a hydrogen bond with the residue of the active PHE140 protease site. The catalytic residues CYS145 and HIS41 represented interactions of the type π, π-π stacked and π-alkyl with the entire beta-carboline group, which was composed of three hydrophobic rings. The remaining residues were of the π-sigma type, hydrogen-carbon acceptors, and halogen acceptors from residues THR25, THR26, as well as other residues from the active site GLU166, GLN189, GLY143, HYS164, respectively.

Ligand ZINC1875405 represented two hydrogen bonding interactions with residues THR25 and PHE140. Additionally, four more polar interactions of the type π-π stacked, π-aquil, aquil and π-sulfur with residues HIS41, MET49, CYS145 and MET165, respectively, were formed. The other interactions were of hydrophobic van der Waals type.

Ligand ZINC2092396 interacted by hydrogen interaction with the residue PHE140, π and π-alkyl with CYS140, π-π stacked and π-alkyl HIS41, and van der Waals with GLN189, GLY143, HIS164, GLU166. Other interactions occurred with hydrogen bonds by the ligand nitrobenzene group with the ASN142 residue and a π-sulfur interaction of the beta-carboline group with MET165 residue.

**Figure 3.**
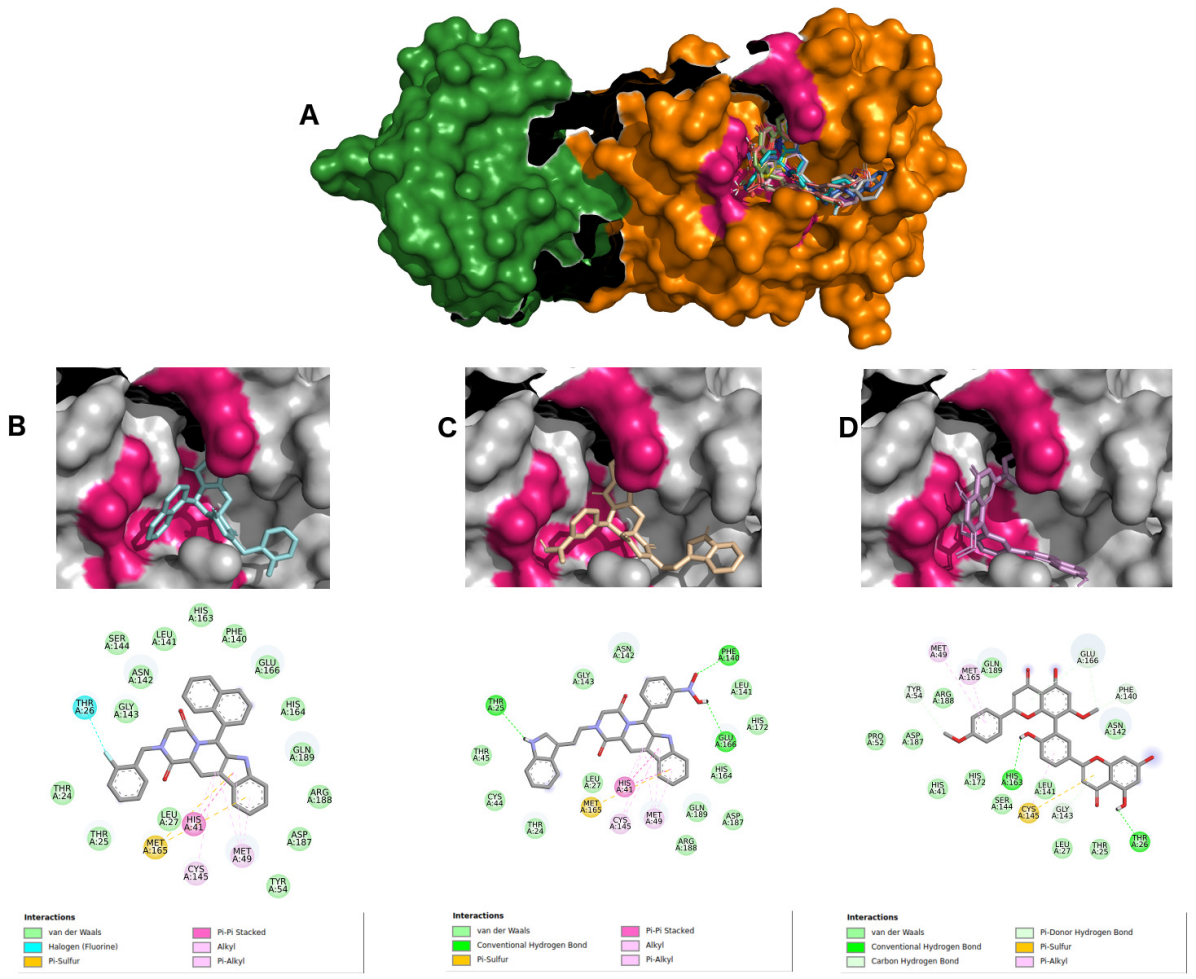
( **A**) SARS-CoV-2 main protease complexed with 10 best hits for remdesivir pharmacophore molecules. Protomer A is represented in green surface, and protomer B in orange surface. ZINC2104424 in cyan ( **B**), ZINC1875405 in wheat ( **C**) and ZINC44018332 in violet ( **D**) inside 6LU7 binding site and their 2D interaction maps with pocket amino acids.

The remdesivir pharmacophore-like search returned two beta-carboline alkaloids (ZINC2104424 and ZINC1875405), as well as one polyflavonoid (ZINC44018332), which interacted with the COVID-19 main protease active pocket showing affinity energies below -10.0 kcal/Mol. Figure 3A–D shows the details of all ligand interactions, as well as the top three molecules interaction maps.

Ligand ZINC2104424 also occupied the region of the active site ( Figure 3B), showing polar interactions π, π-alkyl, π-π stacked and π-sulfur types from beta-carboline with HIS41, MET49, CYS145, and MET165 amino acids. Moreover, an interaction of THR26 halogen with the ligand fluorobenzene group also occurred. Other hydrophobic interactions were van der Waals, mostly with residues of the active site: PHE140, GLY143, HIS163, HIS164, GLU166 and GLN189.

Ligand ZINC1875405 ( Figure 3C) displayed three hydrogen interactions with the indole group, and two oxygen interactions from a nitrobenzene of THR25, PHE140 AND GLN166, respectively. Several van der Waals-type hydrophobic interactions were found with GLY143, HIS164 and GLN189 amino acids. Furthermore, four polar interactions (π, π-alkyl, π-π stacked and π-sulfur) with residues HIS41, MET49, CYS 145 and MET165, respectively, were also retrieved.

Ligand ZINC2092396 ( Figure 3D) exhibited two hydrogen interactions with HIS163 and THR26 by its hydroxyl from the flavonoid nucleus, as well as four more π-donor hydrogen bonding interactions with residues TYR54, PHE140, GLY143 and GLU166. Besides, three π-alkyl and π-sulfur interactions made with MET49, CYS145 and MET165 were also retrieved. Other hydrophobic interactions were of van der Waals type.

**Figure 4.**
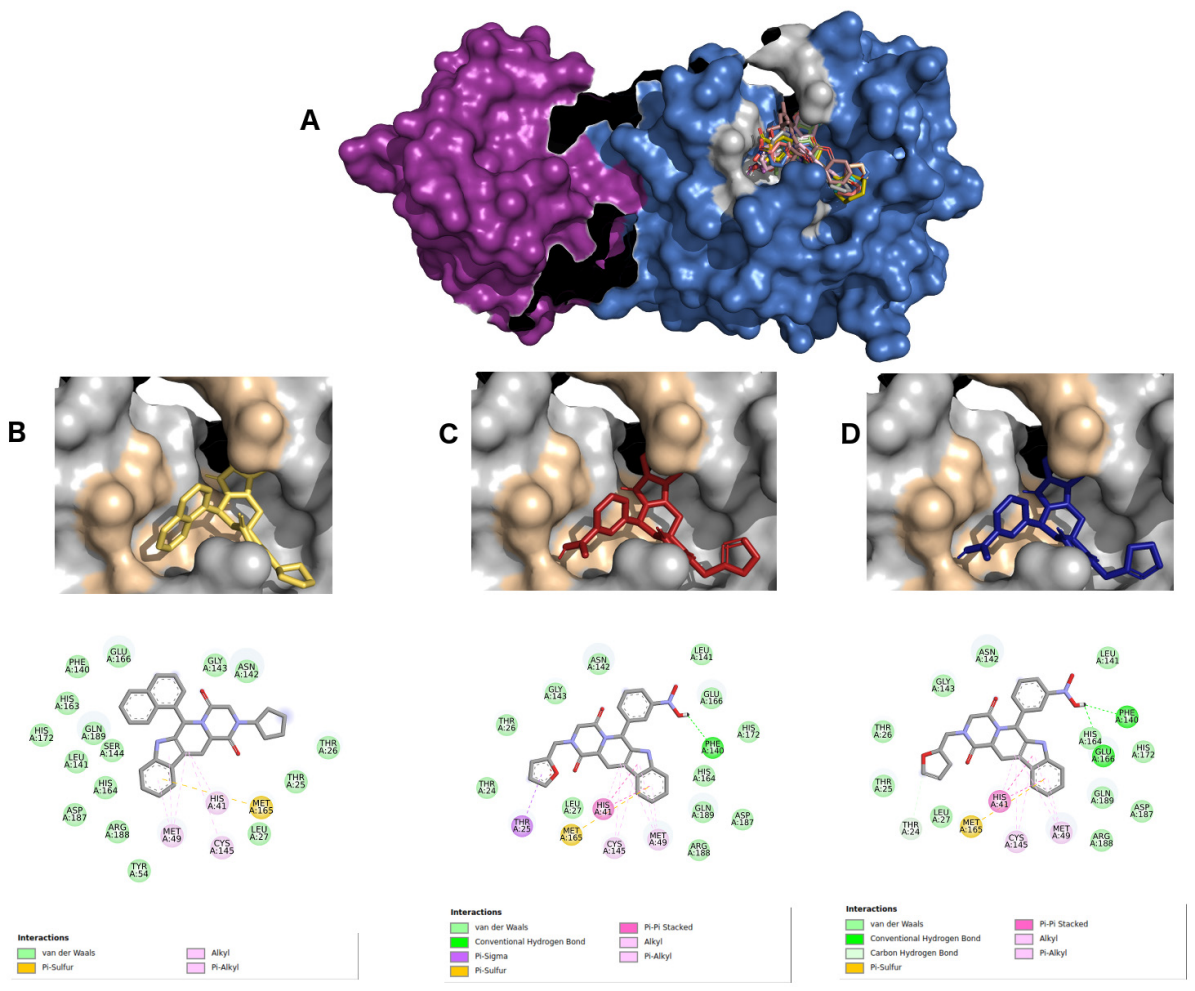
( **A**) Best hits for hydroxychloroquine pharmacophore. Protomer A is represented using a violet surface, and protomer B in marine blue surface. ZINC2101723 in yellow ( **B**), ZINC2094526 in red ( **C**), and ZINC2094304 in dark blue ( **D**) inside 6LU7 binding site and their 2D interaction maps with pocket amino acids.

Figure 4 shows the interactions between 6LU7 active sites and the three best hits from derived molecules of hydroxychloroquine pharmacophore (ZINC2101723, ZINC2094526, ZINC2094304). These complexes displayed affinity energies varying from -10.2 kcal/Mol to -9.6 kcal/Mol, and all the ligands were classified as beta-carboline alkaloid derivatives.

The beta-carboline group of the ligand ZINC2101723 ( Figure 4B) formed four π-alkyl, alkyl and π-sulfur type interactions with HIS41, MET49, CYS145 and MET165 residues, as well as other hydrophobic interactions from its naphthalene and beta-carboline groups with the active site amino acids PHE140, GLY143, HIS163 E164, GLU166 and GLN189. Ligand ZINC2094526 ( Figure 4C) displayed a hydrogen bond interaction with PHE140 by its nitrobenzene group. Five polar interactions (π-sigma, π-aquil, π-π stacked and π-sulfur) were observed with residues THR25, HIS41, MET49, CYS145 and MET165. For ligand ZINC2094304 ( Figure 4D **)**, two hydrogen bonds with residues PHE140 and GLU166 by its nitrobenzene group were formed. In addition, this ligand formed four polar interactions (π-π stacked, π-alkyl, alkyl and π-sulfur) with residues HIS41, MET49, CYS145 and MET165, respectively. Other van der Waals type interactions could also be identified.

**Figure 5.**
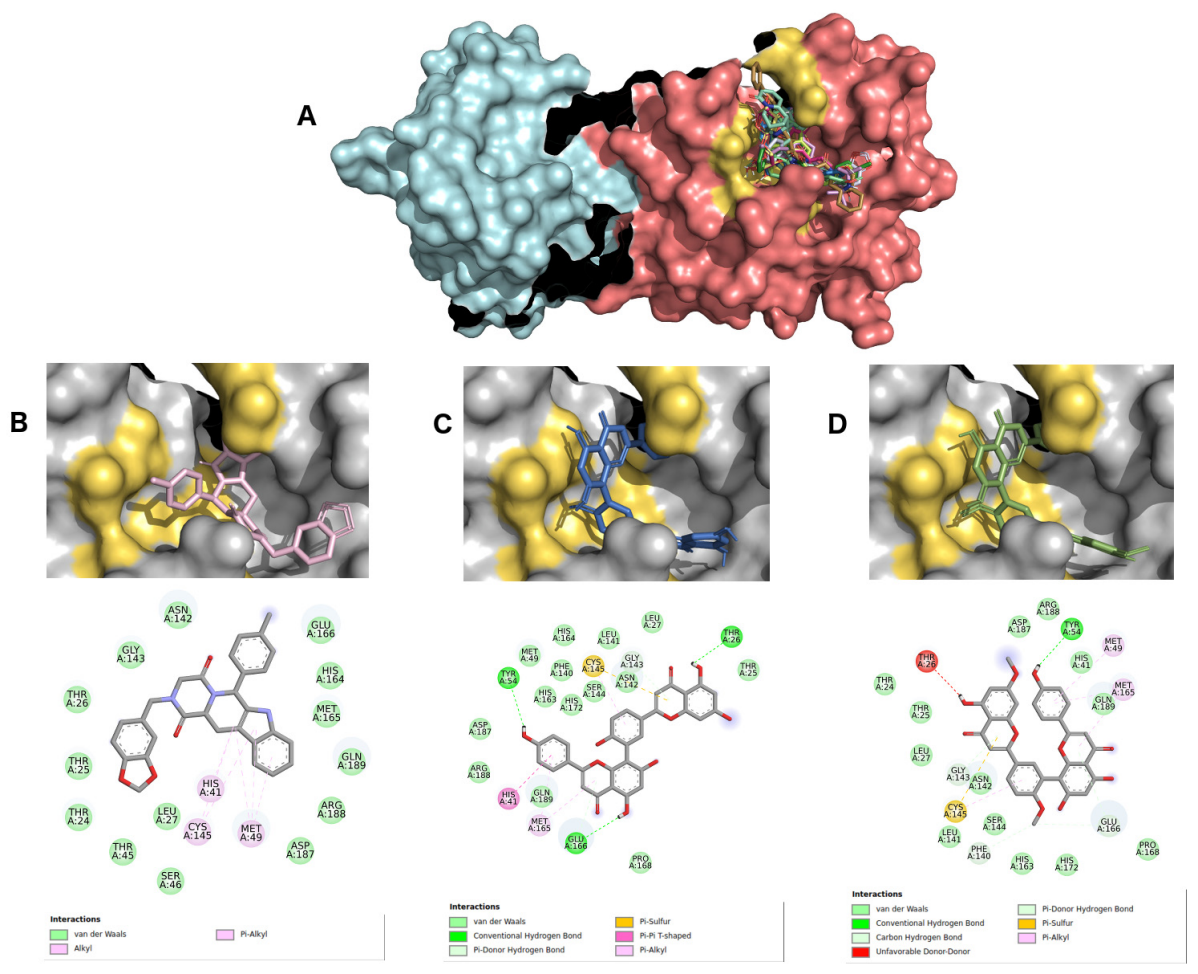
Virtual screening results for the N3 pharmacophore. ( **A**) COVID-19 main protease is represented in cyan (protomer A) and dark salmon (protomer B). The best complexes are formed by the alkaloid ZINC2101723 in pink ( **B**) and two polyflavonoids ZINC2094526 in marine blue ( **C**) and ZINC2094304 in lemon green ( **D**), and their 2D interaction maps with pocket amino acids are shown below each complex.

The N3 pharmacophore revealed one beta-carboline alkaloid (ZINC2101723) and two polyflavonoids (ZINC2094526 and ZINC2094304). This group displayed affinity energies ranging from -9.8 kcal/Mol to -10.1 kcal/Mol. In Figure 5, the best complex interactions with the protease, as well as their positions inside the binding pocket are depicted.

Ligand ZINC2104482 ( Figure 5B) formed a large number of hydrophobic interactions (14 van der Waals interactions), surrounding the active site amino acid, such as GLY143, HIS164, GLU166 and GLN189. Furthermore, this ligand formed three π-alkyl and alkyl bonds with HIS41, MET49, CYS145 residues. Ligand ZINC3984030 ( Figure 5C) exhibited three hydrogen bonds with THR26, TYR54 and GLU166 residues by OH groups of flavonoid nuclei. A π-donor hydrogen bond interaction of the GLY143 residue was also observed. Moreover, three polar interactions (π-π stacked, π-alkyl and π-sulfur) were identified with HIS41, CYS145 and MET165. The rest of the interactions were van der Waals type. Ligand ZINC1531664 ( Figure 5D) showed a hydrogen bond by its OH group TYR54. Additionally, four π-donor hydrogen bond and hydrogen carbon bond interactions with residues PHE140, GLY143, GLU166 also occurred. Two polar interactions of the type π-alkyl and π-sulfur were observed with MET49, CY145, and MET165, and the other hydrophobic interactions were of van der Waals type.

### Re-docking validation experiments

**Figure 6.**
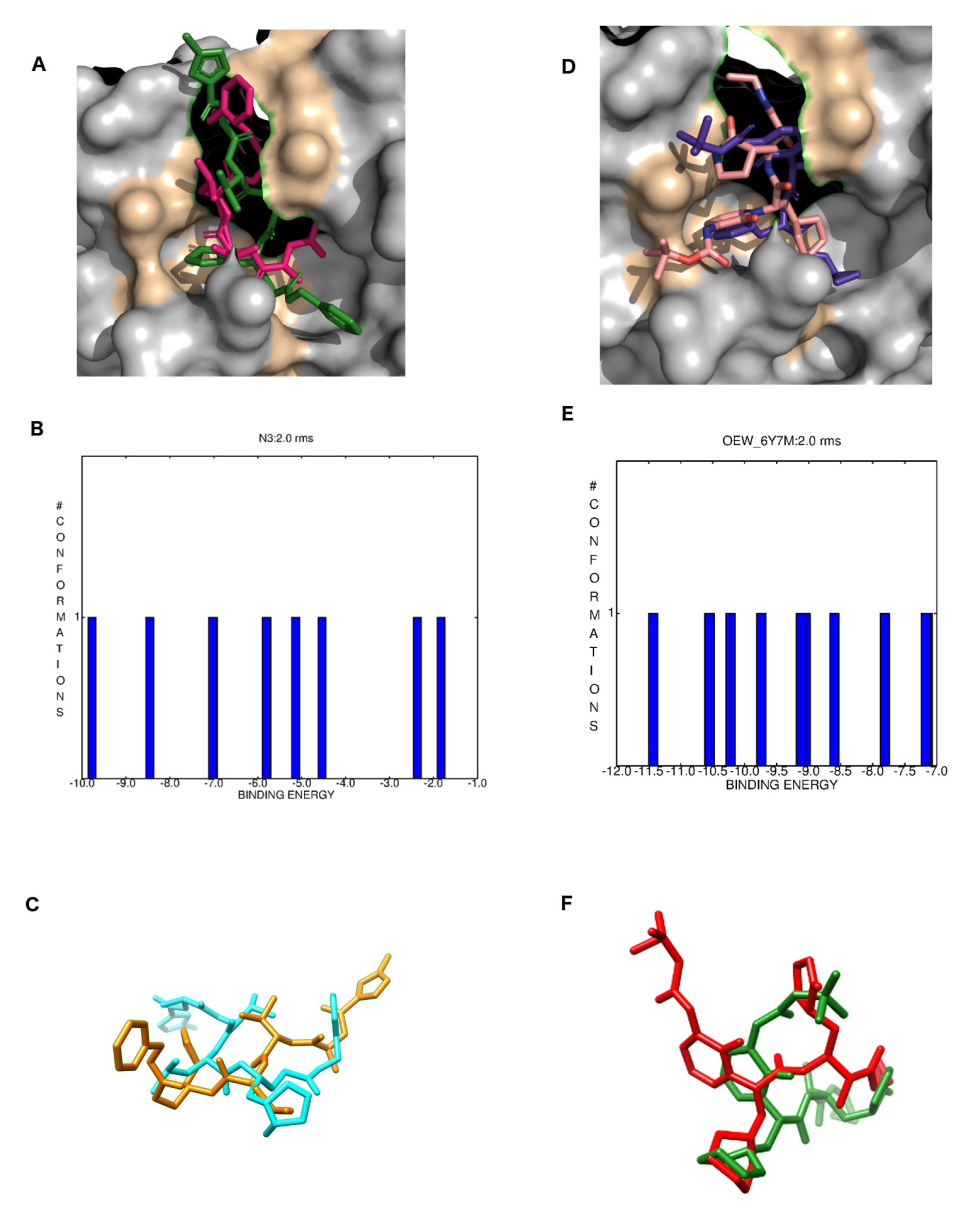
Re-docking validations for N3 and OEW. ( **A**) crystallographic (hot pink) and re-docked (green) N3 inhibitor of the 6LU7 SARS-CoV-2 M ^pro^ inside its binding pocket; ( **B**) N3 docking best clustering conformations; ( **C**) aligned N3 crystallized (yellow) and re-docked (cyan). ( **D**) crystallographic (pink) and re-docked (purple) OEW inhibitor of the 6LU7 SARS-CoV-2 M ^pro^ inside its binding pocket; ( **E**) OEW docking best clustering conformations; ( **F**) aligned OEW crystallized (green) and re-docked (red).

Crystallographic ligands N3 and OWE were re-docked with their respective M ^pro^ structures 6LU7 and 6Y7M. As can be seen in Figure 6A, both N3 and OEW molecules bound into similar positions in comparison to their original crystallographic forms. The Figure 6B depicts the best clustering conformations graph for N3 with a free energy of binding ranging from -1.83 kcal/Mol to -9.7 kcal/Mol. Figure 6C shows the superposition between the crystalized and re-docked N3 structure. Even though N3 is peptide-like with 13 routable bonds, it presented an RMSD of 1.94 Angstroms for its best conformation ( Table 2). OEW re-docking is shown in Figure 6D in the same way as for N3 where both the crystallized and docked structures bound into the same pocket. Conformational population of OEW clustering results returned a free energy of binding ranging from -7.0 kcal/Mol to -11.5 kcal/Mol but, on the other hand, the structure with binding energy of -8.86 kcal/Mol exhibited the smallest RMSD value ( Figure 6E). Additionally, OEW presented an RMSD of 1.97 Å in comparison to its crystallized form ( Figure 6F).

## Discussion

Docking results revealed 39 pharmacophore-like natural ligands, which can be used as drug candidates for inhibiting SARS-CoV-2 main protease activity. Furthermore, we ranked the three best candidates for each known ligand pharmacophore as the best potential drug molecules (and totaling 12 molecules) for *in vitro* and *in vivo* assays purposes, but not excluding the other 28 molecules. For these cases, ligands are included in two most expressive chemical classes: β-carboline alkaloids and polyflavonoids. Additionally, all ligands exhibiting better affinity energies than the known drugs was used as references for construction of pharmacophoric characteristics: OEW ^6^, remdesivir ^48^, hydroxychloroquine ^49^, and N3 ^20^. Furthermore, all of these molecules used as start points for pharmacophore generation were previously reported in docking studies as probable Mpro inhibitors ^50– 53^, as well as in *in vitro* and *in vivo* studies. 

The groups of OEW and hydroxychloroquine pharmacophores presented their three most promising ligands classified as β-carboline alkaloids. This class of molecules is reported by different authors with antiviral activities. According to Gonzalez *et al.* ^45^, β-carboline Alkaloids are widely distributed in nature, and its derivatives exhibited activity against Herpes Simplex Viruses by blocking virus replication. Additionally, Gonzalez *et al.* ^45^ demonstrated the action of these alkaloids in dengue virus RNA replication. Furthermore, several other studies suggest alkaloid activity against viral proteases ^54– 56^. Similarly, remdesivir pharmacophore revealed two β-carboline alkaloids (ZINC2104424 and ZINC1875405). In addition, we detected a polyflavonoid (ZINC44018332) as one probable active molecule from a different class against SARS-CoV-2 main protease, and several authors have already described flavonoid activity as viral protease inhibitors ^57– 59^, as well as antiviral molecules acting in different target classes ^58, 60– 62^. N3 pharmacophore displayed two flavonoids as the best molecules and just one β-carboline alkaloid. These results indicate that both classes of molecules could be explored for *in vitro* and *in vivo* tests to evaluate their potential antiviral activities for not only SARS-CoV-2 but also for other viruses of medical interest.

Other classes of molecules were found in our screening for protease activity that were previously described in antiviral studies: anthracenes ^63^, angular pyranocoumarin ^64, 65^, and flavonoid-3-O-glycoside ^66^. Interaction maps of these complexes are available as *Extended data* ^67^.

All the known ligands (OEW, remdesivir, hydroxychloroquine and N3), which were used for validating our computational screening, exhibited worse affinity energies in docking calculations (ranging from -7.8 kcal/Mol to -5.2 kcal/Mol) than the screened natural compounds (ranging from -10.6 kcal/Mol to -9.1 kcal/Mol). Moreover, all the 40 selected ligands docked inside M ^pro^ active site, as previously described in several antiviral studies, and interacted in the region of connection between domains I and II with amino acids HIS41 and CYS145 ^19, 20, 23, 40, 43, 68^.

Novel M ^pro^ ketoamide inhibitors were recently proposed, including the OEW ligand (ligand 13b) that was used in our study, and the authors detected a reduction in RNA replication in human cells infected with SARS-CoV-2, and also described binding interactions with its main protease. Besides, the same study indicated a ketoamide as a probable drug candidate against this virus ^40^.

In a recent study, authors have proposed the peptidomimetic molecule N3 as a drug candidate against COVID-19, and described its binding interactions with the crystallographic structures of SARS-CoV-2 and other viral proteases. Their study reported that N3 can bind in all the active pockets from the main proteases of HCoV-NL63, SARS-CoV, and MERS-CoV ^69^.

Other molecules have also been tested as antivirals for effectiveness in inhibiting SARS-CoV-2 replication in cell culture. Two drugs exhibited a promising inhibitory effect: remdesivir, an experimental drug developed for the treatment of Ebola virus infection ^43, 70^, and hydroxychloroquine, a drug known for its effectiveness in the treatment of malaria and autoimmune diseases ^43^. Remdesivir is an adenosine triphosphate analogue initially described in the literature in 2016 as a potential treatment for Ebola ^71^, and this drug has been indeed considered as a potential treatment for SARS-CoV2, ^70^. Notably, remdesivir has demonstrated antiviral activity in the treatment of MERS and SARS ^72^ in animal models, both of which are caused by coronaviruses ^73^. Pharmacophore models are widely used in medicinal chemistry with the aim of amplifying the number of drug candidates, and according to this definition, they are represented by a 3D arrangement of abstract features instead of chemical groups ^74^. Remdesivir is a nucleotide analogue with capacity to inhibit RNA polymerase ( Table 1): this molecule displayed almost the same pharmacophoric features ( Figure 1) as for N3 and OEW, and, besides, both of them have already tested experimentally. Additionally, as can be seen in Table 2, the best hit is ZINC1875405, which was found in both OEW and remdesivir pharmacophore searching, and this could be explained by their similar characteristics. Hydroxychloroquine is an aminoquinoline-like chloroquine ^75^. It is a drug commonly prescribed for the treatment of uncomplicated malaria, rheumatoid arthritis, chronic discoid lupus erythematosus, and systemic lupus erythematosus ^76^. Chloroquine and hydroxychloroquine have been investigated for the treatment of SARS-CoV-2 ^77^, and they have been reported to have direct antiviral effects, such as inhibition of flaviviruses, retroviruses (like HIV), and many coronaviruses. Additionally, hydroxychloroquine is capable of inhibiting the zika virus NS2B-NS3 protease, and exhibited good viral replication blocking in infected JEG3 cells in concentration of 80 µM of hydroxychloroquine ^78^. Furthermore, the use of chloroquine and its analogues can be corroborated by a recent study showing that, with EC _50_ of 1.13 µmol/L and selectivity index (SI) greater than 88, chloroquine can effectively inhibit SARS-CoV-2 at the cellular level ^79^. Its effectiveness in the human body for SARS-CoV-2 infection; however, has not yet been clinically proven. Another *in silico* study with chloroquine detected its interactions with viral NSP-3B type protease ^80^.

The co-crystallized molecules N3 and OEW are both peptide analogs, which presented RMSD values of 1.94 Å and 1.97 Å in re-docking experiments, respectively. Generally, docking validations protocols use co-crystallized ligands, to test the accuracy of the program to predict the correct ligand docking poses in comparison to known conformations, and its RMSD varies 1.5 or 2 Å depending on ligand size for being considering acceptable ^81^. The number of studies using protein-peptide docking has been increasing rapidly, followed by the number of applied drug design programs and models. On the other hand, the use of RMSD validations with experimental structures is not always the best criterion of docking success, once it can be influenced by resolution quality, as well as the number of peptide residues ^82^. Thus, we can consider that N3 and OEW docking validations are in acceptable RMSD ranges.

## Conclusions

In our study, we compared the pharmacophores of four well-tested human coronavirus (including SARS-Cov-2) main protease drug candidates to 50,000 structures of natural compounds from the ZINC Database. The three best molecules selected for each pharmacophore class are mainly classified as β-carboline alkaloids, and polyflavonoids. The best ligand-SARS-CoV-2 complexes exhibited better affinity energies in comparison to drug molecules used in this study. Furthermore, all the screened molecules bonded between domains -I and -II and formed interactions with the catalytic residues HIS41 and CYS145 in similar positions as previously described from other authors in viral protease inhibitor studies. Altogether, we propose these compounds as possible SARS-CoV-2 protease inhibitors, which can be used for subsequent *in vitro* and *in vivo* tests for finding novel drug candidates.

## Data availability

### Source data

Structures of natural compounds were downloaded from the ZINC Database.

Crystal structures of COVID-19 main protease were downloaded from the Protein Data Bank, accession numbers 6LU7 (in complex with N3) and 6Y7M (with OEW).

### Extended data

Harvard Dataverse: Replication Data for: Computational screening for potential drug candidates against SARS-CoV-2 main protease. https://doi.org/10.7910/DVN/GYFXA0 ^67^.

This project contains the following extended data:

- 2D interaction maps of all OEW pharmacophore-like ligands (PNG).
- 2D interaction maps of all Remdesivir pharmacophore-like ligands (PNG).
- 2D interaction maps of all Hydroxychloroquine pharmacophore-like ligands (PNG).
- 2D interaction maps of all N3 pharmacophore-like ligands (PNG).

Extended data are available under the terms of the Creative Commons Zero "No rights reserved" data waiver (CC0 1.0 Public domain dedication).
