# Supplementary material for: Computational screening for potential drug candidates against the SARS-CoV-2 main protease
Source: F1000Res. 2020 Dec 21;9:ISCB Comm J-514. Originally published 2020 Jun 4. [Version 2] doi: 10.12688/f1000research.23829.2 (PMC7780344; doi:10.12688/f1000research.23829.2)
Supplement: Peer review history

# Peer review: Reviewer response for version 2

**DOI:** 10.5256/f1000research.31458.r76341

**Published:** 2020-12-22

**Authors:** Murtaza M. Tambuwala

**Affiliations:** 1 School of Pharmacy and Pharmaceutical Sciences, Ulster University, Coleraine, UK

This revived version is Acceptable for publication

Is the work clearly and accurately presented and does it cite the current literature?

Partly

If applicable, is the statistical analysis and its interpretation appropriate?

Not applicable

Are all the source data underlying the results available to ensure full reproducibility?

Partly

Is the study design appropriate and is the work technically sound?

Yes

Are the conclusions drawn adequately supported by the results?

Yes

Are sufficient details of methods and analysis provided to allow replication by others?

Yes

Reviewer Expertise:

Inflammation, cancer, and infections research

I confirm that I have read this submission and believe that I have an appropriate level of expertise to confirm that it is of an acceptable scientific standard.

# Peer review: Reviewer response for version 2

**DOI:** 10.5256/f1000research.31458.r76340

**Published:** 2020-12-22

**Authors:** Alaa Aljabali

**Affiliations:** 1 Department of Pharmaceutics and Pharmaceutical Technology, Yarmouk University, Irbid, Jordan; 2 Yarmouk University, Irbid, Jordan

Thanks for addressing my comments I am happy with the revised version and recommend accepting the article in its current form

Is the work clearly and accurately presented and does it cite the current literature?

Yes

If applicable, is the statistical analysis and its interpretation appropriate?

Not applicable

Are all the source data underlying the results available to ensure full reproducibility?

Yes

Is the study design appropriate and is the work technically sound?

Yes

Are the conclusions drawn adequately supported by the results?

Yes

Are sufficient details of methods and analysis provided to allow replication by others?

Yes

Reviewer Expertise:

viral nanotecnology, biotechnology and drug delviery

I confirm that I have read this submission and believe that I have an appropriate level of expertise to confirm that it is of an acceptable scientific standard.

# Peer review: Reviewer response for version 1

**DOI:** 10.5256/f1000research.26292.r75869

**Published:** 2020-12-09

**Authors:** Alaa Aljabali

**Affiliations:** 1 Department of Pharmaceutics and Pharmaceutical Technology, Yarmouk University, Irbid, Jordan; 2 Yarmouk University, Irbid, Jordan

The manuscript is well written, balanced, and follows the scientific logic and flow very nicely. The work is very novel and holds great potential. The approach is very useful and holds great potential in using the docking system to maybe identify new antiviral candidates in this pandemic.

 Some minor comments:

1. Please list the software used for generating the docking work.
2. Could you please improve the figure axis of 6 bars for clarity (B and E)?
3. Could you please add a short paragraph on the novelty of this approach of molecular docking?
4. Could you please indicate if any of the reported drugs have been used in clinical trials originated from the docking approach?
5. Would it be possible to show the pharmacophore score for both acceptors and donners for ZINC? 

Some key reference should be included in the manuscript:

1. COVID-19 pandemic: an overview of epidemiology, pathogenesis, diagnostics and potential vaccines and therapeutics ^1^.
2. COVID-19: Underpinning Research for Detection, Therapeutics, and Vaccines Development ^2^.
3. The Structural Basis of Accelerated Host Cell Entry by SARS-CoV-2 ^3^.
4. Questions concerning the proximal origin of SARS‐CoV‐2 ^4^.

Is the work clearly and accurately presented and does it cite the current literature?

Yes

If applicable, is the statistical analysis and its interpretation appropriate?

Not applicable

Are all the source data underlying the results available to ensure full reproducibility?

Yes

Is the study design appropriate and is the work technically sound?

Yes

Are the conclusions drawn adequately supported by the results?

Yes

Are sufficient details of methods and analysis provided to allow replication by others?

Yes

Reviewer Expertise:

viral nanotecnology, biotechnology and drug delviery

I confirm that I have read this submission and believe that I have an appropriate level of expertise to confirm that it is of an acceptable scientific standard, however I have significant reservations, as outlined above.

## References

[1] : COVID-19 pandemic: an overview of epidemiology, pathogenesis, diagnostics and potential vaccines and therapeutics. *Ther Deliv*.11(4) : 10.4155/tde-2020-0035 245-268 10.4155/tde-2020-0035 32397911PMC7222554

[2] : COVID-19: Underpinning Research for Detection, Therapeutics, and Vaccines Development. *Pharm Nanotechnol*.2020;8(4) : 10.2174/2211738508999200817163335 323-353 10.2174/2211738508999200817163335 32811406

[3] : The Structural Basis of Accelerated Host Cell Entry by SARS-CoV-2†. *FEBS J*.2020; 10.1111/febs.15651 10.1111/febs.15651 33264497PMC7753708

[4] : Questions concerning the proximal origin of SARS‐CoV‐2. *Journal of Medical Virology*.2020; 10.1002/jmv.26478 10.1002/jmv.26478 PMC789891232880995

# Response

**Published:** 2020-12-15

**Authors:** Bruno Andrade

**Affiliations:** Universidade Estadual do Sudoeste da Bahia, Brazil

The manuscript is well written, balanced, and follows the scientific logic and flow very nicely. The work is very novel and holds great potential. The approach is very useful and holds great potential in using the docking system to maybe identify new antiviral candidates in this pandemic.

***We would like to thank you very much for your description of our work. We improved the paper based on your recommendations.***

 Some minor comments:

 Please list the software used for generating the docking work.

***The docking protocol is described in the Methods section (Docking Studies subsection)***

 Could you please improve the figure axis of 6 bars for clarity (B and E)?

***Figure 6 (B and E) is part of Autodock 4 results, visualized at Autodock Tools program. This program doesn’t give us a good resolution docking energy graph. We improved the figure quality using an image editor program in this new version. Thank you for your suggestion.***

 Could you please add a short paragraph on the novelty of this approach of molecular docking?

***Our focus in this work was using pharmacophore modeling in order to find new possible ligand Mpro inhibitors, as well as repurpose approved drugs for SARS-CoV-2. In this case, we used a common docking approach for confirming our pharmacophore predictions. All docking protocol is described in the Methods section. Thank you very much for your suggestion.***

 Could you please indicate if any of the reported drugs have been used in clinical trials originated from the docking approach?

***All molecules used for pharmacophore generation were tested in vitro, in vivo, and in silico. We included four citations for the in silico tests in the discussion section.***

 Would it be possible to show the pharmacophore score for both acceptors and donners for ZINC?

**We used OpenEye vROCS program, which considers the Tanimoto Combo (TC) scores for selecting the most similar pharmacophore-like molecules. We included a column in table 2, showing the TC score for each best-selected ligand. Thank you very much for this suggestion.**

 Some key reference should be included in the manuscript:

 COVID-19 pandemic: an overview of epidemiology, pathogenesis, diagnostics and potential vaccines and therapeutics1.

**We included the suggested reference in this new version. Thank you.**

 COVID-19: Underpinning Research for Detection, Therapeutics, and Vaccines Development2.

 The Structural Basis of Accelerated Host Cell Entry by SARS-CoV-23.

 Questions concerning the proximal origin of SARS‐CoV‐24.

**We included the suggested reference in this new version. Thank you.**

 References included

 1.        Amawi H, Abu Deiab GI, A Aljabali AA, Dua K, Tambuwala MM. COVID-19 pandemic: An overview of epidemiology, pathogenesis, diagnostics and potential vaccines and therapeutics. Therapeutic Delivery. 2020.

 2.        Aljabali AAA, Bakshi HA, Satija S, Metha M, Prasher P, Ennab RM, et al. COVID-19: Underpinning Research for Detection, Therapeutics, and Vaccines Development. Pharm Nanotechnol. 2020;

 3.        Hagar M, Ahmed HA, Aljohani G, Alhaddad OA. Investigation of some antiviral N-heterocycles as COVID 19 drug: Molecular docking and DFT calculations. Int J Mol Sci. 2020;

 4.        C S, S DK, Ragunathan V, Tiwari P, A S, Brindha Devi BD. Molecular docking, validation, dynamics simulations, and pharmacokinetic prediction of natural compounds against the SARS-CoV-2 main-protease. J Biomol Struct Dyn. 2020;

 5.        Naik VR, Munikumar M, Ramakrishna U, Srujana M, Goudar G, Naresh P, et al. Remdesivir (GS-5734) as a therapeutic option of 2019-nCOV main protease–in silico approach. J Biomol Struct Dyn. 2020;

 6.        Peele KA, Potla Durthi C, Srihansa T, Krupanidhi S, Ayyagari VS, Babu DJ, et al. Molecular docking and dynamic simulations for antiviral compounds against SARS-CoV-2: A computational study. Informatics Med Unlocked. 2020;

# Peer review: Reviewer response for version 1

**DOI:** 10.5256/f1000research.26292.r75868

**Published:** 2020-12-09

**Authors:** Murtaza M. Tambuwala

**Affiliations:** 1 School of Pharmacy and Pharmaceutical Sciences, Ulster University, Coleraine, UK

"Computational screening for potential drug candidates against the SARS-CoV-2 main protease" is overall a timely interesting review focused on COVID pandemic.

 However, since the literature on COVID research is being updated so rapidly this review seems to have used older references and some latest and relevant papers have been missed out. 

 Below I have provided suggestions for several such manuscripts that need to be included and discussed:

- The Structural Basis of Accelerated Host Cell Entry by SARS-CoV-2 ^1^.
- The Importance of Research on the Origin of SARS-CoV-2 ^2^.
- Questions concerning the proximal origin of SARS‐CoV‐2 ^3^.
- Probing 3CL protease: Rationally designed chemical moieties for COVID-19 ^4^.
- COVID-19 pandemic: an overview of epidemiology, pathogenesis, diagnostics and potential vaccines and therapeutics ^5^.
- COVID-19: Underpinning Research for Detection, Therapeutics, and Vaccines Development ^6^.

I am confident that this review will attract a good level of readership from both the public can the research community.

Is the work clearly and accurately presented and does it cite the current literature?

Partly

If applicable, is the statistical analysis and its interpretation appropriate?

Not applicable

Are all the source data underlying the results available to ensure full reproducibility?

Partly

Is the study design appropriate and is the work technically sound?

Yes

Are the conclusions drawn adequately supported by the results?

Yes

Are sufficient details of methods and analysis provided to allow replication by others?

Yes

Reviewer Expertise:

Inflammation, cancer, and infections research

I confirm that I have read this submission and believe that I have an appropriate level of expertise to confirm that it is of an acceptable scientific standard.

## References

[1] : The Structural Basis of Accelerated Host Cell Entry by SARS-CoV-2†. *FEBS J*.2020; 10.1111/febs.15651 10.1111/febs.15651 33264497PMC7753708

[2] : The Importance of Research on the Origin of SARS-CoV-2. *Viruses*.2020;12(11) : 10.3390/v12111203 10.3390/v12111203

[3] : Questions concerning the proximal origin of SARS‐CoV‐2. *Journal of Medical Virology*.2020; 10.1002/jmv.26478 10.1002/jmv.26478 PMC789891232880995

[4] : Probing 3CL protease: Rationally designed chemical moieties for COVID-19. *Drug Dev Res*.2020; 10.1002/ddr.21724 10.1002/ddr.21724 32729640

[5] : COVID-19 pandemic: an overview of epidemiology, pathogenesis, diagnostics and potential vaccines and therapeutics. *Ther Deliv*.11(4) : 10.4155/tde-2020-0035 245-268 10.4155/tde-2020-0035 32397911PMC7222554

[6] : COVID-19: Underpinning Research for Detection, Therapeutics, and Vaccines Development. *Pharm Nanotechnol*.2020;8(4) : 10.2174/2211738508999200817163335 323-353 10.2174/2211738508999200817163335 32811406
